# Treatment of Radiation-Associated Dysphagia with Statins (TRADstat): protocol for a prospective, single-site, single-arm pilot trial

**DOI:** 10.1136/bmjopen-2026-125077

**Published:** 2026-10-20

**Authors:** Jacqui Frowen, Danny Rischin, Sandro Porceddu, Marliese Alexander, Mitchell Dyer, Haris Ahmad, Tsien Fua, Juliana Di Iulio, Angela Pizzolla, Karla Gough

**Affiliations:** 1 Nutrition and Speech Pathology Department, Peter MacCallum Cancer Centre, Melbourne, Victoria, Australia; 2 Sir Peter MacCallum Department of Oncology, The University of Melbourne, Melbourne, Victoria, Australia; 3 Department of Medical Oncology, Peter MacCallum Cancer Centre, Melbourne, Victoria, Australia; 4 Department of Radiation Oncology, Peter MacCallum Cancer Centre, Melbourne, Victoria, Australia; 5 Pharmacy Department, Peter MacCallum Cancer Centre, Melbourne, Victoria, Australia; 6 Clinical Research Development & Operations, Peter MacCallum Cancer Centre, Melbourne, Victoria, Australia; 7 Department of Cancer Research, Peter MacCallum Cancer Centre, Melbourne, Victoria, Australia; 8 Department of Nursing, The University of Melbourne, Melbourne, Victoria, Australia

**Keywords:** Adult radiotherapy, Head & neck tumours, Fibrosis, Speech pathology, Cancer Survivors

## Abstract

**Introduction:**

Radiation-associated dysphagia (RAD) is one of the most common and profound side effects of radiotherapy for head and neck cancer (HNC), but there is currently no effective treatment for persistent or late-onset RAD. Pravastatin has shown potential in reversing established fibrosis through modulation of the connective tissue growth factor signalling pathway and decreasing pro-inflammatory and profibrotic cytokines. Treatment of RAD with statins (TRADstat) is a pilot trial designed to evaluate the feasibility (primary outcome), efficacy potential, acceptability and safety of a 12-month trial of pravastatin as an antifibrotic agent for managing late-RAD.

**Methods and analysis:**

48 patients who are at least 2 years post (chemo)radiotherapy for HNC of the oropharynx, nasopharynx, hypopharynx or larynx and have at least moderate dysphagia will be recruited to this single-site, single-arm pilot trial. Participants will receive pravastatin 40 mg/day continuously for up to 12 months. Primary feasibility outcome data (recruitment, trial retention and medication adherence) will be summarised as a proportion with 95% CI. These will be judged against pre-specified criteria for trial progression and analysed using a one-sided exact binomial test. All remaining data will be analysed descriptively.

**Ethics and dissemination:**

The trial protocol and informed consent documents have been reviewed and approved by the Peter MacCallum Cancer Centre Human Research Ethics Committee (HREC/121555/PMCC, protocol version 1.3, 11 May 2026). Study findings will be disseminated through peer-reviewed publications, at national and international conferences and at key stakeholder forums.

**Trial registration number:**

clinicaltrials.gov NCT07217938

**STRENGTHS AND LIMITATIONS OF THIS STUDY**

- Rigorous study design with comprehensive safety monitoring and a wide range of outcome measures (feasibility, acceptability, clinician-rated function and patient-reported outcomes)
- Includes pre-specified criteria that require all feasibility benchmarks to be met before progressing to a definitive trial without amendments.
- Carefully considered sample size that accounts for all primary feasibility outcomes, potential efficacy signals and anticipated attrition.
- While the sample size was boosted to examine efficacy potential, this may be complicated by sample heterogeneity (ie, variability in time since treatment).

## Introduction

 Therapeutic approaches to head and neck cancer (HNC) continue to evolve, with advances in diagnostic techniques and multimodality treatment, an increasing proportion of human papillomavirus-related disease with improved prognosis and the growing role of immunotherapy in recurrent or previously untreatable disease.^1–3^ These changes in the HNC landscape mean that more patients are not only surviving the disease but also living with the often devastating long-term effects of treatment.

Radiation-associated dysphagia (RAD) is one of the most common and profound side effects of radiotherapy (RT) for HNC, affecting up to 70% of patients.^4–6^ Caused primarily by tissue fibrosis, RAD is complex and insidious and can result in aspiration pneumonia, malnutrition, long-term tube feeding, poor quality of life (QoL), anxiety and depression.^7–9^ Much of the research to date has focused on acute RAD—during and in the first 6–12 months after treatment^10^ with many studies investigating approaches to prevent the development of RAD through prophylactic exercises. However, compliance is poor,^11^ and long-term benefits remain unclear.^11 12^ RAD is also recognised as a debilitating late effect of treatment, with up to 39% of patients experiencing ‘severe’ late RAD that manifests years into survivorship.^6 9 13 14^ Our group recently completed a large cross-sectional study of patient-reported swallowing outcomes on 626 patients who had been treated between 6 months and 10 years previously. Dysphagia was reported by 444 (71%) patients and poor swallowing-related QoL by 138 (22%), with issues being reported irrespective of time since treatment.^15^

Late RAD is caused primarily by progressive radiation-induced tissue fibrosis or scarring,^16 17^ in which the usually supple and dynamic muscles of the pharynx are slowly replaced with atrophied, immobile tissue characterised by poor vascular supply and increased collagen deposits.^16 18^ Late RAD has so far proven refractory to intervention,^19–21^ with management limited to compensatory approaches and patient and carer support and education. Two recently completed trials have taken a physical rehabilitation approach to treat late RAD. The MANTLE pilot trial established the feasibility and safety of manual therapy to improve cervical range of motion and functional outcomes in long-term survivors of HNC with late RAD.^22^ While improvements were seen in several secondary endpoints, swallowing outcomes showed minimal to no measurable effect, and the study was not powered to assess efficacy. The HIT-CRAD trial is a three-arm multicentre randomised controlled trial (RCT) in patients at least 6 months post chemo-RT. Patients were randomised to either strengthening exercises, strengthening exercises and functional swallowing therapy, or strengthening exercises, functional swallowing therapy and non-invasive brain stimulation. Primary swallowing outcomes have not yet been published, but positive adherence and feasibility have been reported,^23^ and although swallowing outcomes were worse for patients who were >5 years post chemo-RT, they still showed comparable and significant improvements after 8 weeks of rehabilitation.^24^

### Trial rationale

Pravastatin, a class of statins (ie, 3-hydroxy-3-methylglutaryl-coenzyme A reductase inhibitors), has the potential to prevent the development of fibrosis by inhibiting the excessive extracellular matrix deposition that causes fibrosis.^25^ Promising results in vitro and in vivo following radiation to intestinal and lung tissue^26 27^ and skin^28^ indicate that pravastatin may prevent fibrosis development when given prior to the commencement of RT. Preclinical models have also identified a potential role in reversing established fibrosis through modulation of the connective tissue growth factor signalling pathway involved in fibrosis development and maintenance^29^ and decreasing pro-inflammatory and profibrotic cytokines.^30 31^ On the basis of these promising preclinical results, the phase 2 trial ‘Pravacur’ published in 2019 investigated the use of pravastatin as an antifibrotic agent in patients with established cutaneous and subcutaneous radiation-induced fibrosis of the neck following HNC treatment.^29^ The single-arm trial of 60 patients demonstrated that pravastatin was well tolerated and reduced objective and patient-reported fibrosis severity in 36% and 50% of patients, respectively, after 12 months. QoL also significantly improved. Pravastatin was well tolerated, with a low occurrence of toxicities. As the pathogenesis of internal fibrosis is believed to be the same as external (cutaneous/subcutaneous) fibrosis,^32^ we hypothesise that a similar improvement in internal fibrosis and subsequently swallowing function may be observed in patients with late RAD who receive a 12-month course of pravastatin. Pravastatin was selected to closely replicate and build on the findings of the Pravacur study,^29^ which used pravastatin based on earlier preclinical evidence, including its absence of tumour protection and its ileal specificity.^25 26^ Feasibility and acceptability of the treatment must first be established, in addition to gathering preliminary evidence of efficacy potential that could help inform a future definitive trial.

### Potential benefits and significance

This trial is the first step in finding a novel solution to a currently untreatable condition that can have devastating effects on a person’s health and QoL. An effective treatment would have the potential to improve QoL, reduce or eliminate feeding tube use, the need for nutritional supplements, medical risks associated with dysphagia and healthcare costs. The proposed intervention is simple, cost-effective and accessible. If proven feasible, with adequate acceptability, tolerability and safety, a subsequent definitive trial would be developed, with the aim of confirming the off-label use of this safe and inexpensive medication. Such treatment would be accessible to thousands of patients worldwide.

### Aims and objectives

This trial is conducted to examine the feasibility, acceptability and potential efficacy of a 12-month trial of pravastatin as an antifibrotic agent for the management of dysphagia and dysphagia-related outcomes in patients previously treated with RT for HNC.

The co-primary objectives are to assess feasibility of recruitment, trial retention, medication adherence, and to gather preliminary evidence on efficacy potential after 12 months of pravastatin.

Secondary objectives are to assess the acceptability of the intervention and trial procedures, and to assess the tolerability and safety of pravastatin in this population.

Exploratory objectives are to describe changes in patient-reported and clinician-rated measures of swallowing problems and patient-reported symptoms and impacts of head and neck lymphoedema and fibrosis after 12 months of pravastatin; examine any evidence of re-emergence of symptoms (‘rebound effect’) 6 months after ceasing pravastatin; and examine the relationship between dysphagia-related outcomes and immune-related biomarkers.

## Methods and analysis

### Study design

TRADstat is a prospective, single-site, single-arm pilot trial (figure 1). Given that this is an initial evaluation of pravastatin for reversing internal fibrosis and the typically refractory nature of late fibrotic effects, a single-arm design including tolerability and safety assessments is deemed appropriate. This protocol has been written in accordance with the Standard Protocol Items: Recommendations for Interventional Trials (SPIRIT) statement^33^ and Consolidated Standards of Reporting Trials (CONSORT) extension to pilot and feasibility studies.^34^

**Figure 1:**
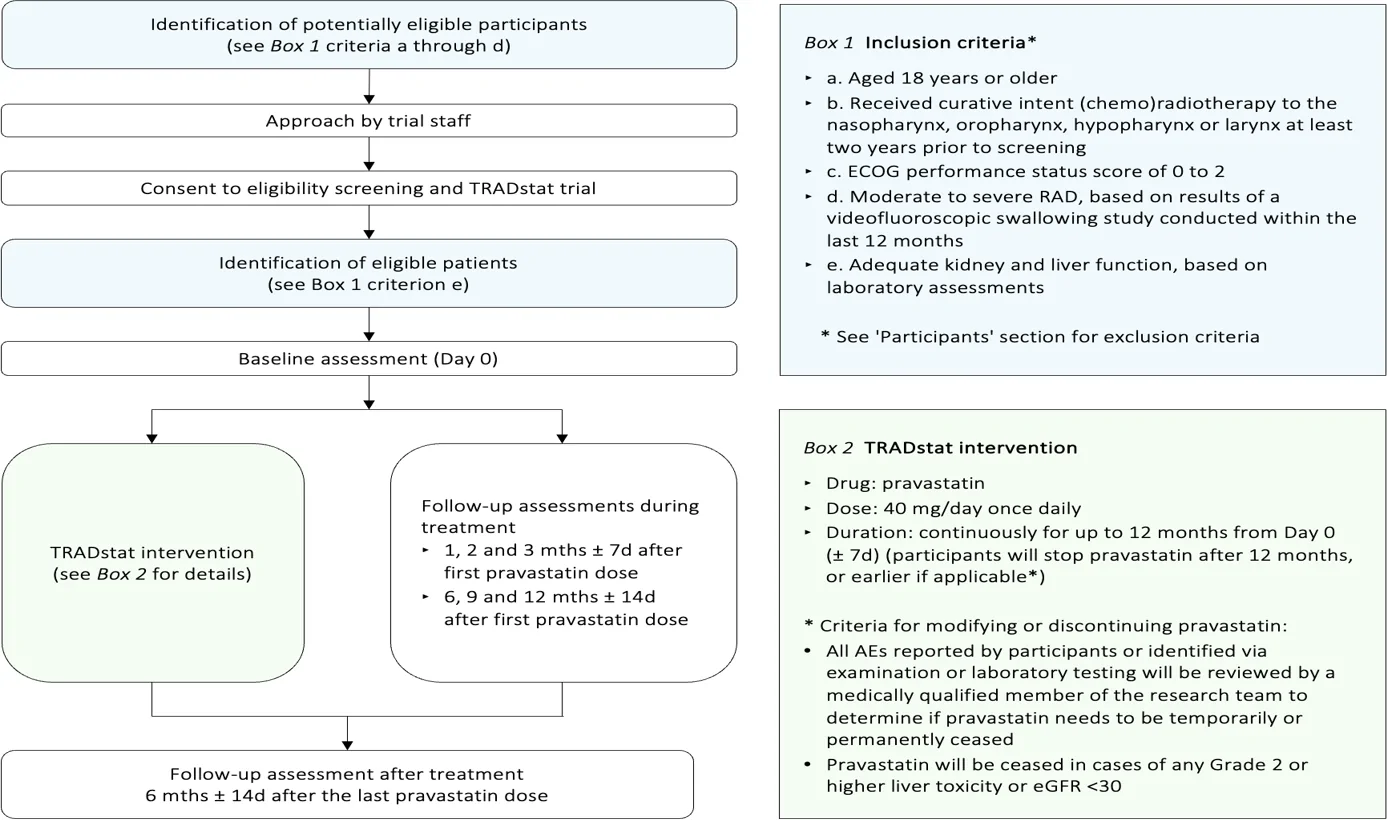
TRADstat trial schema. AEs, adverse events; ECOG, Eastern Cooperative Oncology Group; eGFR, estimated glomerular filtration rate; RAD, radiation-associated dysphagia; TRADstat, Treatment of Radiation-Associated Dysphagia with Statins.

### Trial setting and participants

Potential participants will be identified through the outpatient clinics at the Peter MacCallum Cancer Centre. Specifically, patients who are referred to or attend medical, nursing or allied health clinics and report persistent or worsening dysphagia will be considered for review of eligibility and screening. Patients will also be identified through cross-sectional studies being conducted concurrently at our centre on a similar cohort of patients.

Eligibility criteria for participants are as follows: aged ≥18 years; received curative intent chemo-RT to the nasopharynx, oropharynx, hypopharynx or larynx at least 2 years prior to screening; had moderate to severe RAD, defined as Dynamic Imaging Grade for Swallowing Toxicity version 2 (DIGEST_v2_) grade ≥2 or chronic penetration-aspiration (PAS) score >3 identified on a videofluoroscopic swallowing study (VFSS) conducted within the last 12 months; had adequate kidney function, defined as estimated glomerular filtration rate (eGFR) ≥40 mL/min; adequate hepatic function, defined as aspartate aminotransferase (AST) and alanine aminotransferase (ALT) levels less than two times the upper normal limit (ULN) and bilirubin level <1.5 times the ULN (unless patient has Gilbert’s syndrome with isolated bilirubin elevation); and Eastern Cooperative Oncology Group (ECOG) Performance Status score of 0–2.

Patients will be excluded if they have known hypersensitivity to pravastatin and/or any excipients, myasthenia, history of head or neck surgery (other than post-treatment neck dissection) or known active malignancy; are currently taking statins, long-term steroids or fusidic acid; have a history of severe heart failure, muscle toxicity during previous treatments with fibrates or statins, or hereditary muscle diseases; have known medical condition(s) that may impact swallowing function (eg, stroke, neurological conditions and tracheostomy); and/or are pregnant or breastfeeding.

### Intervention

All patients will receive pravastatin 40 mg/day one time per day continuously for up to 12 months. This dose and duration have been chosen based on the success of the Pravacur study.^29^ Patients will stop pravastatin after 12 months of treatment, or earlier if applicable (see ‘Safety and adverse events’), and be re-assessed 6 months following cessation for rebound effects. However, patients identified with above normal cholesterol at baseline will be advised to liaise with their general practitioner (GP, primary care physician) regarding the appropriateness of continuing pravastatin after 12 months for their health.

### Screening and baseline evaluations

All patients who meet trial eligibility criteria will be recorded in an eligibility log and invited to participate in the study. Numbers of patients who decline to participate and reasons for declining (if provided) will be recorded to allow documentation of recruitment and consent rates. Patients who agree to participate and provide written informed consent will undergo baseline assessments. Data to be collected at baseline include demographic, medical background, cancer and cancer treatment information, vital signs, baseline symptoms and ECOG performance status (table 1). Baseline symptoms include any physical symptoms or abnormalities (in addition to dysphagia) regularly experienced by the patient to assist in determining whether subsequent adverse events (AEs) are pre-existing or new since starting pravastatin.

**Table 1:** Schedule of assessments for the TRADstat trial

Assessment	Screening*		Baseline	During treatment				After treatment
	Stage 1	Stage 2						
	Within 28 days of consent		Day 0	1, 2 and 3 mths (± 7 days) after the first pravastatin dose	6 months (± 14 days) after the first pravastatin dose	9 months (± 14 days) after the first pravastatin dose	12 months (± 14 days) after the first pravastatin dose	6 months (± 14 days) after the last pravastatin dose
**Demographic and clinical data**								
Background information†	X							
ECOG performance status	X							
Concomitant medications	X			X	X	X	X	
Laboratory assessments‡		X	X	X	X		X	
Vital signs§			X					
Baseline symptoms			X					
Weight and feeding tube use			X		X		X	X
Adverse events assessment				X	X	X	X	
Pravastatin adherence				X	X	X	X	
**Clinician-rated measures**								
Videofluoroscopy swallowing study (DIGEST_v2_, PAS and CASM)	X		X¶		X		X	X
Performance Status Scale for Head and Neck Patients			X		X		X	X
Functional Oral Intake Scale			X		X		X	X
Trismus			X		X		X	X
**Patient-reported outcome measures**								
Eating Assessment Tool			X		X		X	X
M.D. Anderson Dysphagia Inventory			X		X		X	X
Head and Neck Lymphedema and Fibrosis Symptom Inventory			X		X		X	X
Theoretical Framework of Acceptability Questionnaire							X	X

* Stage 1: screening for potential eligibility; stage 2: screening to confirm eligibility.

† Background information: sex, postcode, language, level of education, smoking and alcohol at time of diagnosis, tumour site, TNM stage, p16 status, radiation dose/fraction, chemotherapy regimen (if applicable), neck radiation laterality, mean radiation dose (to pharyngeal constrictors, parotids and larynx), time since treatment completion, all active medical conditions and prior medical conditions considered relevant.

‡ Laboratory assessments: biochemistry at baseline, 1, 3, 6 and 12 months after the first dose of pravastatin; haematology at baseline, 3 and 12 months after the first dose of pravastatin; lipid profile and research blood samples (optional) taken at baseline and 12 months after the first dose of pravastatin.

§ Vital signs: respiratory rate, heart rate, blood pressure, oxygen saturation (by pulse oximeter) and temperature.

¶ Does not need to be repeated if already completed for clinical assessment within 12 weeks of baseline.

ECOG, Eastern Cooperative Oncology Group; DIGEST, Dynamic Imaging Grade of Swallowing Toxicity; PAS, Penetration-Aspiration Scale; CASM, Computational Analysis of Swallowing Mechanics.

### Study evaluations

Primary feasibility outcomes will be collected throughout the trial and assessed on trial completion using operational data recorded in the TRADstat trial database. All remaining outcome data will be gathered in accordance with the schedule of assessments outlined in table 1.

#### Primary feasibility outcomes

Primary feasibility outcomes include recruitment (the percentage of eligible patients invited to participate who consent and are registered), trial retention (the percentage of registered patients who complete the 6-month follow-up assessment) and medication adherence (percentage of registered patients who remain on pravastatin for 12 months). Pravastatin treatment adherence will be determined through drug accountability processes (drug receipt, dispensing and returns) and via patient report by recording a number from 0 to 7 when asking patients at each time point: “In the past 7 days, how many days did you miss taking pravastatin?”). Trial data will be judged against pre-specified progression criteria (table 2); moving forward to the definitive trial without amendments is contingent on all three measures meeting the desired levels.

**Table 2:** Pre-specified progression criteria for primary feasibility outcomes

Outcome	Progression criteria*	
	Go: proceed to definitive trial	Stop: do not proceed
Recruitment	≥70%	≤40%
Retention	≥80%	≤50%
Adherence	≥80%	≤50%

* Amend criteria if the estimate for each outcome falls between the lower limit of the ‘go’ zone and the upper limit of the ‘stop’ zone, a definitive trial may proceed following consideration of amendments.

#### Efficacy potential

Efficacy potential will be assessed using the DIGEST_v2_. While progressing to a definitive trial is contingent on feasibility outcomes only, a decrease of at least 1 point from baseline after 12 months of pravastatin will provide preliminary evidence of efficacy potential in individual participants. DIGEST_v2_ is a validated method to grade the severity of pharyngeal swallowing impairment using a decision tree to summarise the patterns of penetration/aspiration (safety) and pharyngeal residue (efficiency) observed on the VFSS.^35^ The safety profile is derived by assigning the maximum PAS score observed across a series of standard bolus trials with modifiers to account for the frequency and quantity of penetration/aspiration events. The efficiency profile is assigned through estimation of the maximum percentage of pharyngeal residue also with modifiers to account for variations across bolus types. The combination of safety and efficiency profiles derives an overall severity grade of impairment on a 5-point scale aligned with the Common Toxicity Criteria for Adverse Events version 5.0 (CTCAE v5.0) toxicity framework, so the grade ranges from ‘0’ (normal) to ‘4’ (life threatening or profound impairment). DIGEST_v2_ is an appropriate measure for efficacy potential in this trial, as it is validated on an HNC population,^35^ has excellent inter-rater and intra-rater reliability^36^ and has proven sensitivity to longitudinal changes after head and neck RT^37^ and swallowing therapy.^38^

#### Secondary outcomes

*Acceptability*. Two sets of six items from the generic Theoretical Framework of Acceptability (TFA) questionnaire were adapted to evaluate the healthcare intervention (pravastatin), along with aspects of trial methods and procedures.^39 40^ These items reflect the following TFA constructs: affective attitude, burden, intervention coherence, perceived effectiveness, self-efficacy and general acceptability. Items have five response levels ranging from ‘1’ to ‘5’ with verbal labels appropriate for each item (eg, strongly dislike to strongly like and strongly disagree to strongly agree). Responses to items measuring TFA constructs, except the general acceptability item, are summed and then divided by the number of items to obtain the total mean score. The total score and responses to the general acceptability item may be used as measures of acceptability, with higher scores representing greater acceptability.

*Tolerability*. The percentage of patients who cease pravastatin (due to either patient or clinician choice) because of pravastatin-related toxicity will provide a measure of tolerability. Pravastatin cessation related to other causes will also be reported.

*Safety*. The percentage of patients who experience grade ≥2 toxicity, according to the CTCAE v5.0, and is related (possibly, probably or definitely) to pravastatin will provide a measure of safety. Toxicity will be established through AE reporting according to the schedule outlined in table 1. Clinical AEs will be assessed via patient consult with an experienced nurse or medical practitioner. AEs identified via laboratory assessments include biochemistry (creatine kinase (CK), eGFR, ALT, AST, alkaline phosphatase, gamma-glutamyl transferase, serum bilirubin, total protein and albumin) and haematology (haemoglobin, haematocrit, red blood cells, white blood cells, neutrophils, basophils, eosinophils, lymphocytes, monocytes and platelets).

#### Exploratory outcomes

*VFSS* will be completed according to the DIGEST_v2_ protocol using standard swallows of thin liquid, pudding and cracker boluses.^41^ Digital recordings will be rated by an experienced speech pathologist blinded to the clinical history and study timepoint, except at baseline where many VFSSs will have already been conducted and rated by the treating clinician, which may include study investigators. Ratings will be completed for the measures: DIGEST_v2_ (as previously described), PAS^42^ and Computational Analysis of Swallowing Mechanics (CASM).^43 44^ PAS is a reliable and valid measure used throughout dysphagia research and clinical practice to measure the presence and degree of airway penetration or aspiration. It comprises an 8-point rating scale ranging from 1 (material does not enter the airway) to 8 (material enters the airway, passes below the vocal folds and no effort is made to eject). Higher scores reflect more severe penetration or aspiration. CASM assesses multiple components of swallowing mechanics through coordinate mapping of muscle function during swallowing. It involves 12 coordinates obtained from the lateral view of a VFSS study to map hyoid movement, laryngeal elevation, pharyngeal shortening, tongue base retraction and head and neck extension and pharyngeal constriction. Patient-specific CASM is performed by using a semi-automated tracker tool to map coordinates in every frame of the VFSS recording at 30 frames/s.

*Performance Status Scale for Head and Neck (PSS-HN*) is used to rate a patient’s functional status in key areas affected by HNC and its treatment.^45^ The scale comprises three subscales: normalcy of diet, which measures the extent to which a person can eat a normal diet without restrictions or modifications; eating in public, which measures the person’s comfort and ability to eat in social settings; and understandability of speech, which measures how well the person can be understood by others when speaking. Each subscale is scored from 0 to 100, with higher scores indicating better performance. The PSS-HN has been shown to discriminate levels of functioning across a range of patients with HNC and has demonstrated good inter-rater reliability and sensitivity to change over time.^46^

*Functional Oral Intake Scale (FOIS*) is a 7-point scale that measures the degree to which a person needs to modify the consistency or texture of food they eat and/or requires tube feeding. The scale score ranges from ‘7’ (total oral intake with no restrictions) to ‘1’ (nothing by mouth) and has demonstrated validity and reliability.^47^

*Trismus*. Maximal–interincisal opening (MIO) will be measured in millimetres using the Therabite range of motion ruler. The distance between the edges of the maxillary and mandibular central incisors will be measured. For patients who are edentulous/partially dentate, measurement will be taken from the edentulous ridge to the opposing arch/incisor vertically.

*Eating Assessment Tool (EAT-10*) is a 10-item Likert-type scale that provides a measure of swallowing problems.^48^ Items have five response levels, ranging from ‘0’ (no problem) to ‘4’ (severe problem). Responses are summed to obtain a total score ranging from 0 to 40, with higher scores reflecting more severe swallowing problems. Scores ≥3 represent ‘abnormal swallowing’ in the general population. EAT-10 has been translated into several different language versions including Chinese (Mandarin) and Vietnamese.^49^

*M.D. Anderson Dysphagia Inventory (MDADI*) is a 20-item Likert scale that provides a measure of swallowing-related QoL.^50^ Items focus on perceived limitations in swallowing and associated impact on daily activities and QoL and have five response levels ranging from ‘1’ (strongly agree) to ‘5’ (strongly disagree), although two items are scored in reverse. A global assessment of QoL is based on a response to the item “My swallowing limits my day-to-day activities”. Emotional, physical and functional subscale scores are calculated by summing responses to relevant items (6, 8 and 5 items, respectively). Subscale scores are summed to create a composite score ranging from 20 to 100. Higher scores reflect better swallowing QoL. In current clinical trials, a composite score <60 represents ‘poor’ swallowing-related QoL, scores between 60 and 79 are considered ‘adequate’ and scores ≥80 are considered ‘optimal’.^51^ MDADI has been translated into Chinese (Mandarin) and validated on patients with HNC.^52^

*Head and Neck Lymphedema and Fibrosis symptom inventory (HN-LEF*) is a 33-item scale that provides a measure of symptom burden and functional loss related to lymphoedema and fibrosis in patients with HNC.^53 54^ Each item is answered in a ‘yes’ or ‘no’ format to indicate whether or not the symptom is present. For any item answered ‘yes’, a Likert scale with five response levels is used to indicate the intensity of the respective symptom. Each item then receives a score ranging from ‘0’ (does not experience the symptom) to ‘5’ (experiences the symptom with severe intensity). The 33 symptoms fall into one of seven subscales: (1) soft tissues and neurologic toxicity, (2) systemic symptoms and social functioning, (3) jaw and oral dysfunction, (4) swallowing and taste changes, (5) body image and sexuality, (6) communication and (7) mucosal irritation. Two scores may be generated for each subscale: the average intensity rating for all symptoms in the subscale and the maximum intensity rating for any single symptom within the subscale, although the average score per subscale is more commonly reported in recent studies.

*Nutritional outcomes*. Weight will be recorded as well as feeding tube use based on patient report (the presence of a feeding tube and the percentage of nutritional requirements taken via feeding tube vs oral intake).

*Research bloods* (optional). Immune-related biomarkers will be collected at baseline and after 12 months of pravastatin. Testing will include a cytokine panel and markers related to extracellular matrix turnover, tissue remodelling and inflammation.^55–57^

#### Additional evaluations

Lipid profile (low-density lipoprotein (LDL), high-density lipoprotein (HDL), triglycerides and total cholesterol) will be collected at baseline and 12 months to determine if the patient needs to consult with their GP prior to coming off pravastatin at the 12-month timepoint. Concomitant medications will be recorded based on self-report, with additional medication reconciliation provided by pharmacy as required, and will include any medication taken within 28 days prior to registration and throughout the treatment period.

#### Follow-up evaluations

Feasibility and acceptability data will be collected 6 months after the last dose of pravastatin (ie, 18 months post baseline, or sooner if the patient discontinued pravastatin prior to 12 months for any reason). Clinical and patient-reported outcomes will also be collected to evaluate rebound effects.

### Safety and adverse events

Pravastatin is considered a well-tolerated medicine by the majority of patients. The common side effect (occurring in 1%–10% of patients) is muscle pain.^58^ Rare side effects (<1% of patients) include severe muscle pain, dizziness, headache, nausea and vomiting, diarrhoea or constipation, indigestion, wind or bloating, stomach pain or discomfort, itching, rashes, pimples, blurred or double vision, sexual dysfunction and bladder problems such as frequent urination.^58^ AEs will be assessed and recorded throughout the trial, according to the schedule in table 1. Any AE reported by a patient or identified via examination or laboratory testing will be reviewed by a medically qualified member of the research team to determine whether AEs are associated with pravastatin, whether pravastatin needs to be temporarily or permanently ceased and whether the patient requires further medical care. Grade 1 laboratory abnormalities that are considered clinically significant must be reported as AEs. All grade ≥2 laboratory abnormalities must be recorded as AEs, regardless of clinical significance. Pravastatin therapy will be discontinued in cases of any grade ≥2 liver toxicity, or eGFR <30.

The following medications may be used *with caution* during the treatment phase owing to their potential interactions with pravastatin: ciclosporin, fibrates, cholestyramine and colestipol, macrolide antibiotics, propranolol, bile acid sequestrants, warfarin or other coumarin anticoagulants, lenalidomide, colchicine and nicotinic acid.^58^ Owing to its high risk of interaction with pravastatin, fusidic acid is *prohibited* during the treatment phase of the trial.^58^

If required, a patient may have dose interruptions of up to 28 days to allow safe use of short-term fusidic acid or for the management of AEs. If pravastatin is discontinued for >28 days, this will be marked as a protocol deviation and reassessed by the research team for either (i) suitability to continue with trial treatment or (ii) transfer of the patient into the follow-up phase.

### Data management

A detailed data management plan has been developed according to institutional policies, procedures and guidelines. Data will be stored in a purpose-built Research Electronic Data Capture (REDCap) database, which maintains a comprehensive audit trail, and all actions taken in the system are listed in a secure, time-stamped, retrievable log. Only unidentified patient data will be entered into the REDCap database, and access to the data will only be granted to persons involved in the TRADstat trial. All data generated will be validated to ensure a high-quality, clean and robust dataset for analysis. The project manager will conduct real-time monitoring for patient eligibility data. Post-entry validation and risk-based monitoring will be conducted quarterly and before any significant analyses are conducted.

### Patient and public involvement

Two patient representatives have been actively engaged since the initial planning stage of this study, prior to the grant application process. They have been part of planning meetings and reviewing relevant documents including the grant application, protocol and informed consent. Their involvement has resulted in additional outcomes of interest, improvement in methodology for measuring effectiveness and improving identification and recruitment strategies. They were actively involved in the adaptation of TFA questionnaire items to balance participant burden with outcomes most relevant to patients. Patient representatives will continue to provide their unique perspective on interpretation of results and dissemination of findings. On completion of the study, these patient representatives will assist with sharing research results within consumer forums and advocacy groups, including advice on what information to share and in what format.

### Statistical considerations

#### Sample size calculation

The target sample was determined based on the multi-criteria approach for testing binary feasibility outcomes,^59^ assuming 90% power and a one-sided α=0.05 exact binomial test, and boosted to assess efficacy potential and account for drop-out. For recruitment, with ‘stop’ and ‘go’ cut-points set at 40% and 70%, respectively, a sample of 25 patients is required. For medication adherence 12 months after commencing pravastatin and trial retention at the 6-month follow-up, with ‘stop’ and ‘go’ cut-points set at 50% and 80%, respectively, a sample of 23 patients is required. For efficacy potential, with ‘stop’ and ‘go’ cut-points set at 5% and 20%, respectively, a sample of 38 patients is required. As such, the target sample is 48 patients; this takes into consideration the sample required for all three primary feasibility outcomes and the assessment of efficacy potential, along with projected drop-out (20%).

#### Data analysis plan

Responses to patient-reported outcome measures (PROMs) and clinician-rated measures will be scored using author guidelines. Standard recodes will be applied for analysis and reporting purposes. DIGEST_v2_ scores at each follow-up will be recoded to discrete variables comprising two categories: improvement (decrease of at least one point from baseline) and stable/deterioration (no change or increase of at least one point from baseline). Missing data will not be imputed.

Counts and percentages will be used to summarise missing data including missing items and forms for each PROM and clinician-rated measure at each assessment. Descriptive statistics (counts and percentages, means and SD, or medians and IQRs, as appropriate) will be used to summarise patient characteristics at enrolment, as well as pravastatin-related toxicities (AEs and laboratory assessments), feeding tube use, provision of any swallowing therapy, PROMs and clinician-rated measure scores at each assessment.

Binary feasibility outcome data will be summarised as a proportion with 95% CI using the exact method. These will be judged against pre-specified progression criteria and analysed using a one-sided α=0.05 exact binomial test to test against being in the ‘stop’ zone, designating unacceptable feasibility. Recruitment data will also be summarised as a rate with 95% CI using the Poisson distribution.

Differences between baseline and follow-up on patient-reported outcome (ie, EAT-10, MDADI and HN-LEF) and clinician-rated measure scores (weight, CASM and trismus) will be summarised as a mean with 95% CI. For PROMs, the average of the paired differences will be compared with estimates of minimal clinically important differences, where available.^60^ Effect size estimates, as described by Kazis *et al*,^61^ will be used to characterise the size of observed differences on all measures. Kazis effect sizes are interpreted as per Cohen’s d.^62^ Analyses will include all available data and be performed in R (reference index version 4.5.1 or higher).

## Ethics and dissemination

The trial protocol and informed consent documents have been reviewed and approved by the Peter MacCallum Cancer Centre Human Research Ethics Committee. Prior to participation, written informed consent will be obtained from eligible participants, and each patient has the right to withdraw consent from further trial participation at any time without prejudice to their medical care. This trial will be conducted in compliance with the principles of the Declaration of Helsinki (2013) and the principles of Good Clinical Practice and the Australian National Statement on Ethical Conduct in Human Research. Study findings will be disseminated through peer-reviewed publications, at national and international conferences and at forums for key stakeholders including patients. Individual de-identified participant data, the data dictionary, and statistical code may be made available on reasonable request to the corresponding author, subject to institutional approvals and applicable privacy and ethical requirements.

## Discussion

The proposed TRADstat pilot study represents an important first step in addressing a significant unmet clinical need by evaluating whether pravastatin—an inexpensive, safe and widely available medication—can be repurposed to treat late RAD. RAD is currently an untreatable condition affecting many survivors after radiotherapy for HNC, with significant physical and psychosocial impacts. Addressing the underlying morphology that causes radiation fibrosis has the potential to either mitigate the development of RAD or reverse its impact in cases where it is already developed. Building on the promising findings of the Pravacur trial in reversing radiation fibrosis, TRADstat will comprehensively assess feasibility, acceptability and safety while generating preliminary data on efficacy potential to inform decisions about progression to a definitive trial using pre-specified criteria. In addition, a carefully considered set of PROMs and clinician-completed measures will be collected and analysed to help determine if they merit inclusion in a definitive trial, if such a trial is warranted.

The proposed intervention is simple, cost-effective and accessible, making it particularly valuable for people in remote or rural communities, where swallowing outcomes after HNC treatment are poorer^5^ and access to allied health or specialist HNC services are lacking.^63^ People from culturally and linguistically diverse backgrounds—common in nasopharyngeal cancer, where >80% of patients are of Chinese or Vietnamese background^64^—are under-represented in clinical trials^65^ and face additional barriers to care,^66^ so simple, accessible and acceptable interventions are essential. Previous interventions for late RAD have not only been unsuccessful, but they may rely on complex, time-intensive or specialised approaches^20 22^ that are inaccessible or infeasible for these more vulnerable populations; TRADstat will endeavour to include these populations within the recruited cohort.

The co-primary objectives for TRADstat are to evaluate the feasibility of conducting a larger definitive trial and obtain preliminary indications of efficacy potential. Feasibility will be assessed across several key domains, including recruitment, trial retention and medication adherence. Specifically, the study will be considered feasible to proceed to a definitive trial if recruitment targets are met, with at least 70% of eligible patients consenting to participate and being successfully registered. In addition, retention rates must demonstrate at least 80% of registered participants completing the 6-month follow-up assessment. Finally, adherence to the intervention will be defined as at least 80% of registered participants remaining on pravastatin for 12 months. Together, these criteria provide a structured framework for determining whether progression to a larger trial is justified.

We also aim to collect preliminary evidence of efficacy potential to assist in the development of a larger multi-centre RCT in the future. As a single-arm trial, any positive clinical outcomes cannot be causally attributed to the intervention. However, improvement in swallowing function beyond 2 years post-treatment is uncommon, with deterioration more typically observed over this period.^6 67 68^ In this context, stability of swallowing function following 12 months of pravastatin may itself represent a clinically meaningful outcome, as it could indicate prevention of further decline. Nonetheless, this possibility cannot be confirmed within the design of TRADstat. Our exploratory objective to examine any evidence of re-emergence of symptoms (‘rebound effect’) 6 months after pravastatin cessation may be affected if patients decide, in consultation with their GP (primary care physician), to remain on pravastatin for management of above normal cholesterol if this was identified at baseline.

Despite these limitations, TRADstat is an important step towards finding an intervention that can potentially change the underlying morphology that causes radiation fibrosis and thus improve swallowing outcomes. If successful, pravastatin has the potential to offer a safe, feasible, low-cost and widely accessible treatment option for HNC survivors.

## Data Availability

Data are available upon reasonable request.
