# Supplementary material for: Treatment of Radiation-Associated Dysphagia with Statins (TRADstat): protocol for a prospective, single-site, single-arm pilot trial
Source: BMJ Open. 2026 Oct 20;16(10):e125077. doi: 10.1136/bmjopen-2026-125077 (PMC13684158; doi:10.1136/bmjopen-2026-125077)
Supplement: Supplementary file 1 [file bmjopen-2026-125077.reviewer_comments.pdf]

PEER REVIEW HISTORY

BMJ Open publishes all reviews undertaken for accepted manuscripts. Reviewers are asked to complete a checklist review form and are provided with free text boxes to elaborate on their assessment. These free text comments are reproduced below.

ARTICLE DETAILS

Title (Provisional)

Treatment of Radiation Associated Dysphagia with Statins (TRADstat): protocol for a prospective, single-site, single-arm pilot trial

Authors

Frowen, Jacqui; Rischin , Danny; Porceddu, Sandro; Alexander, Marliese; Dyer, Mitchell; Ahmad, Haris; Fua, Tsien; Di Iulio, Juliana; Pizzolla, Angela; Gough, Karla

---

VERSION 1 - REVIEW

**Reviewer**	**1**
**Name**	**Shaw, Richard**
**Affiliation**	**Institute of Translational Medicine, University of Liverpool,**
**Date**	**03-Aug-2026**
**COI**	**Also a CI of an antifibrotic trial of a differing IMP in late radiation toxicity after H&N cancer**

Rigorously prepared trial protocol with no major concerns.

However, a few questions arise:

1. Safety / tolerability - The protocol states that <10% pts will experience muscle pain, and other side effects will be rare. However the PRAVCUR trial experienced more toxicities than this in more or less the same setting: 18 of 60 patients stopped their treatments as a result prior to the trial endpoint. How do the investigators comment on this apparent disparity?

2. DIGEST grade of dysphagia is a VF -defined scale used to define eligibility and endpoints. The DIGESTV2 scale has been recently published as an improved /amended version - should this be used or is there a rationale for staying with the original? (Bearing in mind it seems to be that the MDAnderson team have amended their own scale, presumably after further experience in 'real-world' as well as trials patients)

**Reviewer**	**2**
**Name**	**Joseph, Nuradh**
**Affiliation**	**Ministry of Health, Sri Lanka**

**Date**	**21-Aug-2026**
**COI**	**None**

It would have been ideal if this was conducted as a randomized study even if it is still in the feasibility stage. Nevertheless, as a protocol paper this is suitable for publication.

VERSION 1 - AUTHOR RESPONSE

REVIEWER 1

Thank you for your positive comments.

1. *Safety / tolerability - The protocol states that <10% pts will experience muscle pain, and other side effects will be rare. However the PRAVCUR trial experienced more toxicities than this in more or less the same setting: 18 of 60 patients stopped their treatments as a result prior to the trial endpoint. How do the investigators comment on this apparent disparity?*

   We thank the reviewer for highlighting this point. In the PRAVACUR trial, although 18 of 60 patients stopped pravastatin before 11 months of treatment, only 8 of those (13%) were because of pravastatin-related toxicities. The remaining withdrew due to consent withdrawal or patient refusal to continue treatment (n=5), or tumor relapse or death (n=5).

   We have based our safety and tolerability data on the regulator (Therapeutic Goods Administration; TGA) approved product information sheet, as this is the strongest available data, and the data which is used when registering a drug. We have now referenced this source for clarity (see pg19-20; changes using Endnote are not tracked).

2. *DIGEST grade of dysphagia is a VF -defined scale used to define eligibility and endpoints. The DIGESTV2 scale has been recently published as an improved /amended version - should this be used or is there a rationale for staying with the original? (Bearing in mind it seems to be that the MDAnderson team have amended their own scale, presumably after further experience in 'real-world' as well as trials patients)*

   Thank you for picking up on this discrepancy. We confirm that the study is using the DIGEST v2 scale. Our reference to "DIGEST" rather than "DIGESTv2" was an oversight in the manuscript wording rather than a discrepancy in the methodology. To avoid confusion, we have removed the citation to the original 2017 publication at the first mention of the scale and now cite only the revised 2022 publication describing DIGESTv2. We have also updated the manuscript to refer consistently to DIGESTv2 throughout (using the subscript notation adopted in the 2022 publication). Please note, although not reflected in its title, the training manual we have cited (ref 41) does correspond to the DIGESTv2 scale.

REVIEWER 2

It would have been ideal if this was conducted as a randomized study even if it is still in the feasibility stage. Nevertheless, as a protocol paper this is suitable for publication.

Thank you; we agree that a randomized study would have been ideal in many ways, however we ultimately felt that a ‘proof of concept’ approach was most appropriate at this early stage.
